# The genomic determinants of adaptive evolution in a fungal pathogen

**DOI:** 10.1002/evl3.117

**Published:** 2019-05-01

**Authors:** Jonathan Grandaubert, Julien Y. Dutheil, Eva H. Stukenbrock

**Affiliations:** ^1^ Environmental Genomics Group Max Planck Institute for Evolutionary Biology August‐Thienemann‐Str. 2 24306 Plön Germany; ^2^ Christian‐Albrechts University of Kiel Am Botanischen Garten 1–9 24118 Kiel Germany; ^3^ Research group Molecular Systems Evolution Max Planck Institute for Evolutionary Biology August‐Thienemann‐Str. 2 24306 Plön Germany; ^4^ UMR 5554 Institut des Sciences de l'Evolution, CNRS, IRD, EPHE Université de Montpellier Place E. Bataillon 34095 Montpellier France

**Keywords:** Adaptation, evolutionary rates, genome evolution, plant pathogenic fungi, recombination

## Abstract

Unravelling the strength, frequency, and distribution of selective variants along the genome as well as the underlying factors shaping this distribution are fundamental goals of evolutionary biology. Antagonistic host‐pathogen coevolution is thought to be a major driver of genome evolution between interacting species. While rapid evolution of pathogens has been documented in several model organisms, the genetic mechanisms of their adaptation are still poorly understood and debated, particularly the role of sexual reproduction. Here, we apply a population genomic approach to infer genome‐wide patterns of selection among 13 isolates of *Zymoseptoria tritici*, a fungal pathogen characterized by extremely high genetic diversity, gene density, and recombination rates. We report that the genome of *Z. tritici* undergoes a high rate of adaptive substitutions, with 44% of nonsynonymous substitutions being adaptive on average. This fraction reaches 68% in so‐called effector genes encoding determinants of pathogenicity, and the distribution of fitness effects differs in this class of genes as they undergo adaptive mutations with stronger positive fitness effects, but also more slightly deleterious mutations. Besides the globally high rate of adaptive substitutions, we report a negative relationship between pN/pS and the fine‐scale recombination rate and a strong positive correlation between the rate of adaptive nonsynonymous substitutions (ω_a_) and recombination rate. This result suggests a pervasive role of both background selection and Hill‐Robertson interference even in a species with an exceptionally high recombination rate (60 cM/Mb on average). While transposable elements (TEs) have been suggested to contribute to adaptation by creating compartments of fast‐evolving genomic regions, we do not find a significant effect of TEs on the rate of adaptive mutations. Overall our study suggests that sexual recombination is a significant driver of genome evolution, even in rapidly evolving organisms subject to recurrent mutations with large positive effects.

**Impact Summary**

Pathogens adapt rapidly in response to the strong interaction with their host. Understanding the genetic bases of this coevolution is a theoretical challenge with potentially critical economic implications. Although an increasing number of candidate genes playing a role in pathogenicity has been reported to be under strong positive selection in several pathogen species, we know little about the impact on genome evolution of rapid adaptation to the host. In particular, many pathogen species display a large amount of clonality, raising the question of the importance of sexual reproduction in rapid adaptation. In this work, we analyzed complete genome sequences from a population of the fungal pathogen *Zymoseptoria tritici* to understand adaptive evolution and coevolution with its wheat‐host. We aim at understanding how selection impacts the genetic diversity along the genome of this pathogen by studying protein‐encoding genes, which constitute more than half of the total genome of this species. Our results show that effector genes, directly involved in the molecular interactions with the host plant, evolve under a rate of adaptive substitutions more than twice as high as that of other genes. Furthermore, we demonstrate a substantial impact of recombination on both positive and negative selection, that is, the fixation of advantageous and removal of deleterious mutations, respectively. Conversely, we do not find a significant impact of transposable elements on the rate of adaptive mutations, as otherwise often proposed as a driver of plant pathogen adaptation. Our results demonstrate that, even in an organism subject to recurrent mutations with strong positive fitness effects, sexual recombination is a significant driver of genome evolution.

Understanding the molecular basis of species adaptation is a major goal of the study of molecular evolution. Pathogens constitute model species for addressing this issue, as their antagonistic interaction with the host drives a co‐evolutionary dynamics where positive selection recurrently replaces existing alleles in response to allelic changes in the host, a scenario termed arms race evolution (Tellier et al. 2014). Based on the finding of high variability in specific genome compartments of several species, it has been proposed that plant pathogens represent exceptional outliers regarding evolutionary rates (Raffaele and Kamoun 2012; Upson et al. 2018; Frantzeskakis et al. 2019). Furthermore, their life cycles typically combine both asexual and sexual reproduction, raising the question of the importance of meiotic recombination on the rate of adaptation, as it has been evidenced in plant and animal species (Seidl and Thomma 2014).

Sexual reproduction is predicted to increase the rate of reciprocal adaptation in a coevolutionary arms race (McDonald and Linde 2002; Marais and Charlesworth 2003). At the genome level, sexual recombination is predicted to affect the patterns of genetic diversity directly and/or indirectly: because (1) cross‐over events may introduce mutations and (2) repair mechanisms of hetero‐duplexes can be biased (biased gene conversion), recombination can directly shape genetic diversity along the genome (Duret and Galtier 2009). Besides, because of its effect on linkage disequilibrium, recombination counteracts the reduction of diversity at neutral sites due to linkage with loci under selection (Maynard Smith and Haigh 1974; Charlesworth et al. 1993). Furthermore, recombination was shown to improve the efficacy of selection by bringing together advantageous alleles in otherwise distinct genetic backgrounds (Hill and Robertson 1966). Initially studied in model species such as *Drosophila melanogaster* (Campos et al. 2014; Castellano et al. 2016), population genomic analyses of an increasing number of species provided empirical support for these theoretical predictions (Corbett‐Detig et al. 2015; Galtier 2016; Pouyet et al. 2018). However, the relative importance of adaptive evolution and the impact of sexual reproduction on genome evolution remain highly debated topics (Haudry et al. 2008; Jaron et al. 2018; Jensen et al. 2018) that have been addressed only in model species (Corbett‐Detig et al. 2015).

Several fungal pathogen species are outliers regarding recombination rates and genome compactness (Stapley et al. 2017). Because of the predicted strong effect of linked selection, gene‐dense genomes evolving under intense selective pressure represent a population genetic conundrum that may favor the evolution of high recombination rates. Among pathogens, clonality is a widespread strategy, and the sexual stage of many species can be remarkably reduced, if even present (Heitman 2006). Many fungal pathogens have a sexual stage, but there is little direct evidence of its frequency of occurrence, and, therefore, of its impact on genome evolution. The existence of alternative mechanisms to increase the rate of adaptation in these species has therefore been hypothesized. Fungal effector genes, which are encoding proteins that interfere with host defenses and may determine the host range of the pathogen, have been found in several species to associate with repetitive DNA. It has been proposed that repeat‐rich genome compartments provide particularly favorable environments for the rapid evolution of new virulence specificities (e.g., Ma et al. 2010; Spanu et al. 2010; Klosterman et al. 2011; Daverdin et al. 2012; Frantzeskakis et al. 2019). Repetitive DNA may locally increase the mutation rate and contribute to gene duplications and structural variation among alleles (Ohta 2000).

The study of adaptive evolution in microbial pathogen models essentially involved genome scans of genes under positive selection and led to the identification of rapidly evolving genes, typically involved in pathogenicity (Aguileta et al. 2010; Stukenbrock et al. 2011; Wicker et al. 2013; Lo Presti et al. 2015; Silva et al. 2015; Schweizer et al. 2018). While these studies advanced our understanding of the molecular basis of host‐pathogen coevolution, the amount of candidate genes under positive selection is only an indirect indicator of the rate of adaptive evolution in these organisms, which is confounded by the demographic history of the species under study and the statistical methodology used for inferring selection. In order to quantify adaptive evolution in genomes, it is necessary to assess systematically genetic variation among genes. In protein‐coding genes, contrasting synonymous and nonsynonymous polymorphisms provides an estimate of the strength of purifying selection, and comparing these patterns with synonymous and nonsynonymous divergence with another species permit to infer the rate of adaptive substitutions (McDonald and Kreitman 1991; Eyre‐Walker and Keightley 2009; Stoletzki and Eyre‐Walker 2011).

In this study, we use the fungal plant pathogen *Zymoseptoria tritici* as a model to address the impact of recombination on the genetic diversity of a rapidly evolving organism. *Z. tritici* infects wheat and reproduces by the production of asexual spores in infected leaf tissues and by sexual recombination between isolates of opposite mating type (Waalwijk et al. 2002). While sexual reproduction is thought to occur at least once per year, as sexual spores constitute a mean of long‐distance dispersal as well as over‐wintering structures (Eyal 1987; Suffert et al. 2011; Morais et al. 2016), little is known about the number of asexual generations. The genome sequence of the fungus revealed a high gene density, with more than half of the genome residing in exons (Goodwin et al. 2011; Grandaubert et al. 2015). Previous studies based on mating experiments and population genomic data have reported exceptional high recombination rates (∼60 cM/Mb), including intragenic recombination hotspots that underline the alleged key role of recombination in the evolution of this species (Croll et al. 2015; Stukenbrock and Dutheil 2017). The similarity between experimental and population genomic estimates of recombination rates suggests that the effective recombination rate in this species is very high, and that the number of asexual generations must be comparatively low compared to the number of sexual generations.

Indirect estimates of large‐scale ancestral recombination rates along the genome suggested evidence for extensive background selection (Stukenbrock et al. 2011). Comparative population genomic analyses of *Z. tritici* and two closely related species, *Z. pseudotritici*, and *Z. ardabiliae*, used genome‐wide estimates of nonsynonymous and synonymous divergence to quantify positive selection and identify candidate genes (Stukenbrock et al. 2010, 2011).

Here, we specifically address the impact of meiotic recombination on the rate of adaptive evolution at the genome scale and contrast it with other genomic factors such as the occurrence of transposable elements (TEs). We analyze an extended population genomics dataset comprising 1.4 million single nucleotides polymorphisms and infer genome‐wide signals of natural selection among 13 isolates of *Z. tritici* collected from bread wheat in Europe and the Middle East, by contrasting patterns of polymorphism and divergence and modeling of the distribution of fitness effects. We also make use of new fine‐scale recombination maps available for this species and TEs annotations. Our analyses reveal a rate of adaptive substitutions in the genome of *Z. tritici* comparable to that reported in animal species, and a more than twice as high rate in genes encoding putative effectors, as well as a distribution of fitness effects of mutations with large positive selection coefficients. We further report a substantial impact of recombination on the efficacy of both positive and negative selection, suggesting pervasive background selection and Hill–Robertson interference along the genome of this species. Based on these results, we propose that high recombination rates and sexual reproduction in this species are a key component of its high adaptive rate.

## Results and Discussion

We generated a population genomic dataset of 13 *Z. tritici* haploid isolates obtained from different field populations in Europe and Iran (Table S1). Given the vast extent of structural variation in genomes of *Z. tritici* isolates, we de novo assembled and aligned the 13 genomes. After filtering (see Material and Methods), the resulting multiple‐genome alignment of ∼27 Mb (Table S2) comprised a total of 1,489,362 SNPs of which approximately 50% located in protein‐coding regions. The SNP data were used to compute the overall genetic diversity of the sample showing a mean value of π = 0.022 per site. Importantly, the multiple‐genome alignment of de novo assembled genomes a priori contains only alignments of orthologous regions.

### LOCAL RATES OF RECOMBINATION CORRELATE WITH THE STRENGTH OF PURIFYING SELECTION, REVEALING PERVASIVE BACKGROUND SELECTION

**Figure 1:**
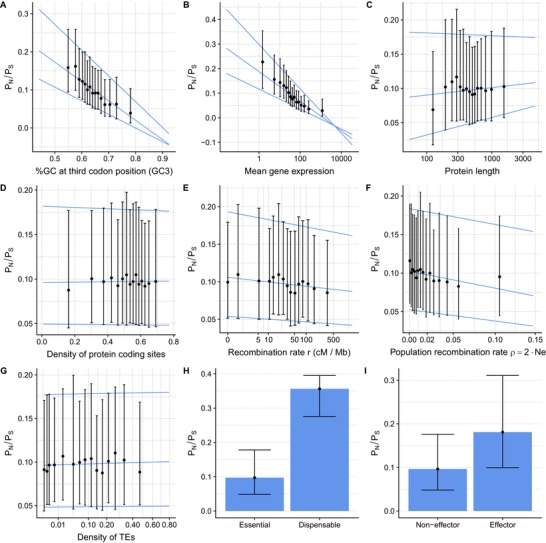
Correlation of the strength of purifying selection with several genomic factors. The pN/pS ratio measures the intensity of purifying selection. (A–G) Points represent median values and error bar the first and third quartiles of the distributions. The *x*‐axis was discretized in categories with equal point densities for clarity of visualization. Lines represent first, median, and third quantile regression on non‐discretized data. (H–I) Colored bars represent median values and error bar the first and third quartiles of the distributions.

We next addressed the genome‐wide strength of purifying selection in protein‐coding genes of *Z. tritici* using the ratio of nonsynonymous to synonymous polymorphisms (pN/pS ratio). We computed pN and pS for each gene as the average pairwise heterozygosity (Romiguier et al. 2014; Ellegren and Galtier 2016). To investigate which genome parameters impact the strength of purifying selection in *Z. tritici*, we compared the pN/pS ratio for each gene to (1) the mean gene expression, (2) the GC content at third codon positions (GC3), (3) the protein length, (4) the local recombination rate, (5) the density in protein‐coding sites, and (6) the density in transposable elements. We used *Z. tritici* gene expression data from early host colonization (four days after spore inoculation on leaves of seedlings of a susceptible wheat host) and in vitro growth (Kellner et al. 2014). Recombination rates were averaged in 20 kb windows and recombination was analyzed independently as *r* (Croll et al. 2015) and *ρ* (Stukenbrock and Dutheil 2017). We further considered whether the gene (7) is an effector candidate and (8) is located on an accessory chromosome (Fig. 1). We restricted our analysis to genes for which pN/pS could be computed (6627 genes, see Materials and Methods) and for which pN/pS was estimated to be <1 (6621 genes).

We identify several variables that significantly impact the strength of purifying selection (summarized in Table 1). Mean gene expression and GC at third codon position have the most substantial effect on pN/pS (Fig. 1A, B), displaying highly significant negative correlations (Kendall's tau = –0.369 and –0.237 respectively, *P*‐values < 2.2.10^−16^ in both cases). Consistent with this observation, studies in yeast and bacteria have previously documented a substantial impact of expression levels on gene evolution whereby highly expressed genes are more conserved reflected as lower pN/pS values (Drummond et al. 2006; Liao et al. 2006). GC3 and mean gene expression are intrinsically highly correlated (Kendall's tau = 0.222, *P*‐value < 2.2.10^−16^), possibly reflecting biases in codon usage whereby optimal codons are GC‐rich at their third position (Fig. S1), as also observed in other organisms (Duret and Mouchiroud 1999). An alternative explanation for the effect of the GC content on pN/pS could be a possible indirect effect of recombination as we also observe a positive correlation of GC3 with the recombination rate (Kendall's tau = 0.097, *P*‐value < 2.2.10^−16^). Recombination and GC3 similarly correlate in other organisms (Duret 2002). In *Saccharomyces cerevisiae*, this correlation has been explained by the impact of biased gene conversion on sequence evolution (Birdsell 2002). However, a thorough search for signatures of GC‐biased gene conversion did not find any pervasive effect of this phenomenon in *Z. tritici* (Stukenbrock and Dutheil 2017). The relationship between pN/pS and GC3 is, therefore, more likely a by‐product of the correlation with gene expression.

**Table 1:** Correlation of pN/pS with genomic factors

Variable	Effect	*P*‐value
GC3	−0.2372	<2.2 × 10^–16^
Expression	−0.3689	<2.2 × 10^–16^
Protein size	0.0227	0.0056
Density of protein coding sites	0.0067	0.4127
Recombination rate (cM/Mb)	−0.0309	1.85 × 10^–04^
Population recombination rate (2.Ne.r)	−0.0394	1.52 × 10^–06^
Density of TEs	−0.0030	0.7197
Effector	0.0845	1.05 × 10^–14^
Dispensable chromosome	0.2590	0.0162

Effects and *P*‐values are calculated using Kendall's correlation of ranks. GC3, GC‐content at third codon positions; TEs, transposable elements.

**Figure 2:**
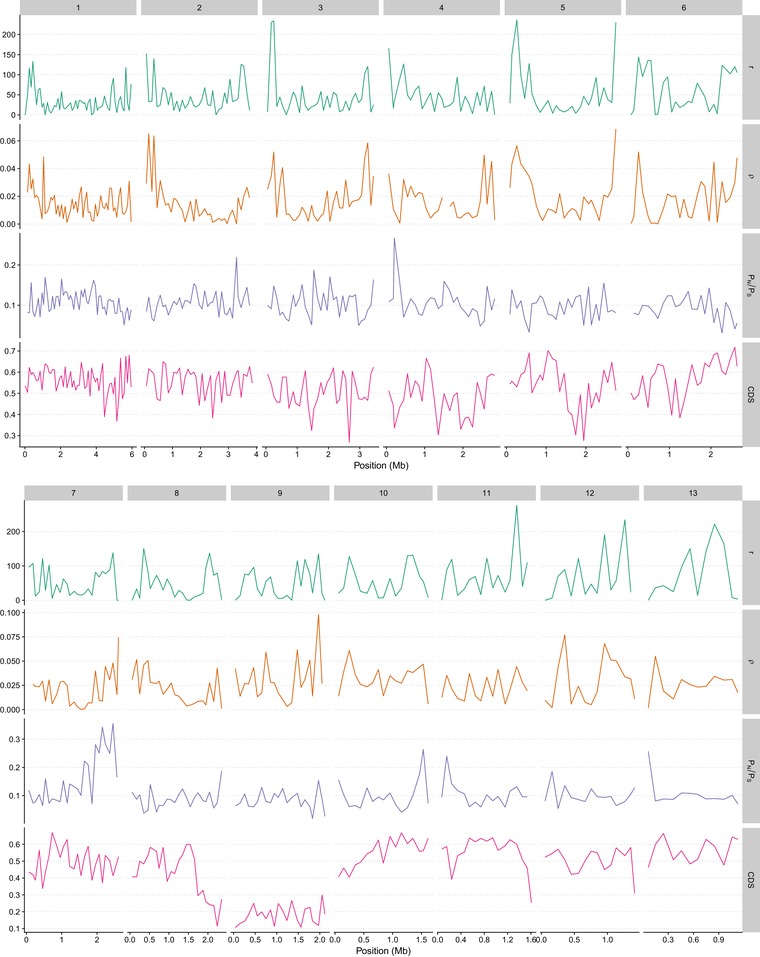
Patterns of selection along the genome of *Z. tritici*. Recombination rate, population recombination rate, pN/pS ratio, and density of coding sites (CDS) are plotted in windows of 100 kb along the 13 essential chromosomes.

Protein size is slightly positively correlated with pN/pS (Table 1), although the effect is due to very short proteins being more conserved. When testing only proteins with >100 amino acids, excluding 348 proteins out of 6621, the correlation is no longer significant (Kendall's tau = 0.012, *P*‐value = 0.1443, Fig. 1C). Coding site density, estimated in 50 kb regions centered on the gene (see Material and Methods), does not have a significant effect on pN/pS (Kendall's tau = 0.0067, *P*‐value = 0.4127, Fig. 1D). Conversely, we observe a significant negative correlation between pN/pS and recombination rate *r* (Kendall's tau = –0.031, *P*‐value = 1.85.10^−4^, Fig. 1E) or *ρ* (Kendall's tau = –0.039, *P*‐value = 1.52.10^−6^, Figs. 1F and 2). These results are in agreement with a model of background selection, where purifying selection at linked loci with low recombination rates reduces the efficacy of selection and allows slightly deleterious mutations to spread more frequently than at loci with high recombination rates (Charlesworth et al. 1993; Nordborg et al. 1996).

Background selection is expected to be stronger in regions with a higher rate of deleterious mutations (Hudson and Kaplan 1995). Assuming that regions with a higher density of coding sites experience more deleterious mutations, we expect the negative correlation between recombination and pN/pS to be more negative in gene‐dense regions. To further test this effect of coding site density, we split our gene set in three subsets of similar numbers of genes according to the density of coding sites. We report that the correlation between r and pN/pS is maximal for the intermediate density class (Kendall's tau = –0.050, *P*‐value = 0.0004927). According to the background selection model, the correlation is lower for the low‐density set (Kendall's tau = –0.030, *P*‐value = 0.03671). It is, however, not significant for the high‐density set, which consists of genes located in regions where more than 58% of the sequence is protein coding (Kendall's tau = –0.013, *P*‐value = 0.3635). We note that, when significant, the effect of coding site density is very weak. The genome of *Z. tritici* is compact and largely uniform regarding gene localization, and the distribution of coding site density is almost normal with a median around 54%. This likely explains why coding site density does not have a strong influence on genetic diversity in this species. Likewise, we do not find a significant effect of the density of TEs on the pN/pS ratio (Table 1 and Fig. 1G).

One part of the *Z. tritici* genome in which recombination is low is the accessory chromosomes (Stukenbrock and Dutheil 2017). This low recombination rate is reflected in our estimates of purifying selection. We find a significantly higher pN/pS ratios in genes located on accessory chromosomes (Wilcoxon rank test, *P*‐value = 0.0162, Fig. 1H), and on the right arm of chromosome 7 (Fig. 2), a genomic region predicted to be an ancestral accessory chromosome fused with a core chromosome (Schotanus et al. 2015). Accessory chromosomes have reduced effective population size due to their presence/absence variation among individuals, resulting in reduced efficacy of selection and a higher pN/pS ratio on these chromosomes.

Finally, we compared the strength of purifying selection of effector and non‐effector genes, and find that genes predicted to encode effector proteins have a significantly higher pN/pS ratio compared to other genes (Wilcoxon rank test, *P*‐value = 1.5 × 10^−14^, Fig. 1I). We speculate that this pattern is due to the fast evolution through positive selection of this particular category of genes, and the higher pN / pS ratio in these genes reflects the less efficient purifying selection at sites linked to positively selected mutations.

These results provide evidence that background selection impacts the genome of *Z. tritici*, and support a central role of recombination in the removal of non‐adaptive mutations in the genome of *Z. tritici* consistent with patterns described in other species such as *D. melanogaster* (Campos et al. 2014).

### EFFECTOR PROTEIN‐ENCODING GENES HAVE A DISTINCT DISTRIBUTION OF FITNESS EFFECTS AND A HIGHER RATE OF ADAPTIVE SUBSTITUTIONS

We aimed to obtain a quantitative assessment of adaptive substitutions in the genome of *Z. tritici*. To this end, we first estimated the nonsynonymous and synonymous divergence dN and dS using a genome alignment of *Z. tritici* and its sister species *Z. ardabiliae*. Furthermore, we used the *Z. tritici* SNP data to compute the unfolded site frequency spectrum (SFS) of synonymous and nonsynonymous sites using *Z. ardabiliae* as outgroup. The synonymous nucleotide diversity was on average over all genes 0.054, reflecting the high diversity in this species. By contrasting divergence and polymorphism data, we estimated the parameters α (proportion of adaptive nonsynonymous substitutions, dN_a_/dN) and ω_a_ (proportion of the dN/dS ratio that is attributable to adaptive mutations, dN_a_/dS; Eyre‐Walker and Keightley 2009). The SFS is strongly affected by demography and the presence of slightly deleterious mutations segregating at low frequencies (Eyre‐Walker and Keightley 2007). State‐of‐the‐art statistical methods account for the latter by modeling the distribution of fitness effects (DFE) of mutations (Gossmann et al. 2010; Galtier 2016). Potential confounding demographic factors such as variable population size, population structure, and linked selection are accounted for by fitting additional parameters to accommodate deviations from a constant size neutral model of evolution. This generic correction assumes that these factors affect both synonymous and nonsynonymous mutations equivalently (Galtier 2016).

We estimated α as well as ω_a_, the rate of adaptive substitutions, using four distinct DFE models accounting for mutations with both slightly deleterious and beneficial effects (see Materials and Methods) and found that the Gamma‐Exponential model best fitted our data (Table 2) in agreement with studies from animals (Galtier 2016). This result suggests the existence of slightly deleterious, as well as slightly beneficial segregating mutations in the genome of *Z. tritici*. When only candidate effector genes are considered (128 genes present in all 13 isolates and for which an outgroup sequence is available), the distinction between models is less pronounced. While Akaike's information criterion (AIC) favors the Scaled Beta model, the Gamma Exponential model offers a comparable fit and leads to very similar estimates of α and ω_a_, and in the following we, therefore, only considered the Gamma Exponential model to ease comparison between gene classes (Table 2). The estimates provide an α value of 35% as a genome average and a ω_a_ value of 0.044. Both values are in the range of what is observed for Mammals (except Primates) but considerably higher than estimates from plants (Gossmann et al. 2010; Galtier 2016). This estimate is consistent with our previous inference of α computed without an underlying DFE model and using only three genome sequences of *Z. tritici* (Stukenbrock et al. 2011). Our previous estimate was an average across all aligned genes and did not distinguish rapidly evolving effector genes. Here, we specifically analyze the subset of candidate effector genes, and we find that the rate of mutations fixed by positive selection is more than twice as high as in non‐effector genes (ω_a_ equal to 0.134 vs. 0.048, Table 2), with 60% of nonsynonymous substitutions in these genes inferred to be adaptive. This average estimate is close to the highest values reported in animals (Galtier 2016) and reflects the strong selective pressure acting on these genes. We note that estimates of α and ω_a_ are slightly higher when only non‐effector genes are used (6639 genes) than when using the complete gene set (6767 genes), a small difference that likely results from sampling variance.

**Table 2:** Estimates of the proportion of adaptive mutation (α) under various models of distributions of fitness effects

Data set	Model	Nb. parameters	Log likelihood	AIC	α	ω_a_
All genes	Neutral	16	−1111.199	2254.398	0.253	0.031
All genes	Gamma	17	−286.142	606.284	0.464	0.058
All genes	GammaExpo	19	−229.425	**496.850**	**0.352**	**0.044**
All genes	DisplacedGamma	18	−286.142	608.284	0.464	0.058
All genes	ScaledBeta	18	−273.334	582.669	0.485	0.060
All genes	BesselK	19	−294.387	626.774	0.514	0.064
Non‐effectors	Neutral	16	−1104.260	2240.519	0.252	0.031
Non‐effectors	Gamma	17	−286.821	607.643	0.463	0.057
Non‐effectors	GammaExpo	19	−230.221	**498.442**	**0.388**	**0.048**
Non‐effectors	DisplacedGamma	18	−286.821	609.643	0.463	0.057
Non‐effectors	ScaledBeta	18	−273.687	583.375	0.458	0.057
Non‐effectors	BesselK	19	−296.728	631.456	0.513	0.063
Effectors	Neutral	16	−101.036	234.071	0.307	0.062
Effectors	Gamma	17	−94.255	222.510	0.485	0.097
Effectors	GammaExpo	19	−87.332	212.664	0.666	0.134
Effectors	DisplacedGamma	18	−94.684	225.369	0.492	0.099
Effectors	ScaledBeta	18	−86.845	**209.689**	**0.600**	**0.120**
Effectors	BesselK	19	−87.416	212.832	0.507	0.102

AIC, Akaike's information criterion; α, proportion of adaptive substitutions; ω_a_, rate of adaptive substitutions. Values in bold indicate the best model fit for each gene set.

**Figure 3:**
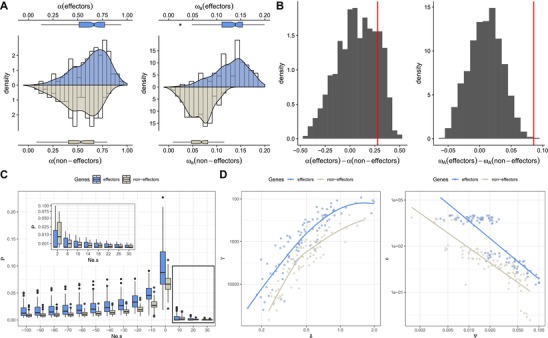
Comparison of the rate of adaptive evolution and distribution of fitness effects in effector and non‐effector genes. (A) Comparison of the estimates of the proportion of adaptive substitutions α and the rate of adaptive substitutions, ω_a_ for genes predicted to encode effector proteins (blue) or not (grey). Histograms (white bars), kernel density plots, and box‐and‐whiskers charts are computed over 100 bootstrap replicates in each case (see Material and Methods). (B) Null distributions of the differences of α and ω_a_ between effectors and non‐effector genes (grey histogram) and the corresponding observed statistics (red line). (C) Average distribution of fitness effects (P), computed as the product of the effective population size Ne and selection coefficient s, over 100 bootstrap replicates for both effector and non‐effector encoding genes. (D) Correlation of inferred parameters over 100 bootstrap replicates of effector and non‐effector encoding genes, for the Gamma (negative selection) and Exponential (positive selection) components, respectively. γ and δ: the mean and shape of the Gamma distribution of negative selection coefficients. ε, mean of the exponential distribution of positive selection coefficients; ψ, the probability that the selection coefficient is positive.

In order to visually compare the two distributions while correcting for the difference in sample size (128 and 6639 genes, respectively), we conducted a bootstrap analysis where we estimated α and ω_a_ in random samples of 128 genes in each category (Fig. 3A). We further assessed the significance of the observed differences with a permutation test with 1000 resampling accounting for the distinct sample sizes (see Methods and Materials). The results reveal that while the differences in α are not statistically significant (*P*‐value = 0.1778), ω_a_ in candidate effector genes is significantly higher (*P*‐value = 0.0020, Fig. 3B). The comparison of the inferred distributions of fitness effects in effector and non‐effector encoding genes reveals an excess of slightly deleterious mutations in genes encoding predicted effector proteins compared to non‐effector genes (Fig. 3C). This means that mutations in the first category of genes are on average less deleterious than in the second (γ = mean Ne.s = –1428 vs. –1547), while beneficial mutations have a larger effect (ε = mean Ne.s = 5227 vs. 0.297) but are less frequent (ψ = probability of having a positive effect = 0.0104 vs 0.0540) in effector‐encoding genes. To assess the robustness of these observed differences, we compared the estimated parameters of the DFE in the 100 bootstrap replicates (Fig. 3D). In agreement with previous reports (Galtier 2016), we find that the mean and shape parameters of the gamma distribution of negative effects, as well as the proportion of positive mutations and their mean effect, are strongly correlated. Despite these intrinsic correlations, the distributions of parameter estimates are distinct and suggest a clear difference between the distribution of fitness effects in the two sets of genes. We note, however, that the set of genes in our dataset is biased toward the more conserved ones, as they have to be present in all 13 isolates and the outgroup species to be included in the analysis. This bias is likely to be stronger in effector‐encoding genes set, and the reported differences in adaptive rates are, therefore, potentially underestimated.

### RECOMBINATION CONTRIBUTES TO HIGH RATES OF ADAPTIVE EVOLUTION IN *Z. tritici*

We next tested whether recombination significantly impacts adaptive evolution in *Z. tritici*. Previous inference of recombination maps in *Z. tritici* based on experimental crosses and population genomic data revealed exceptionally high rates of recombination in this species (Croll et al. 2015; Stukenbrock and Dutheil 2017). To assess the role of recombination in the adaptive evolution of *Z. tritici*, we used the recombination maps generated in these previous studies. Genetic maps resulting from crossing experiments allow inference of the recombination rate *r* (measured as cM/Mb) but are limited in resolution. Conversely, linkage disequilibrium‐based maps generated from population genomic data offer an improved resolution, but only allow inference of *ρ* = 2.Ne.r, where Ne is the effective population size. As such, *ρ* is a proxy for *r* that is affected by both selection and demography. We clustered all analyzed genes according to their *r* and *ρ* values and estimated α and ω_a_ for each case using the gamma‐exponential distribution of fitness effects. In order to assess the variance of our estimates and their robustness to the sampled genes, we further conducted a bootstrap analysis in which we sampled genes in each category 100 times. We report a significant positive correlation between α (averaged over 100 bootstrap replicates) and *r* (Kendall's tau = 0.320, *P*‐value = 0.002978) and ω_a_ and *r* (Kendall's tau = 0.298, *P*‐value = 0.005822). We observe similar correlations when *ρ* is used instead of *r*, or when effector genes are discarded (Supporting Information). These results suggest that a higher recombination rate favors the fixation of adaptive mutations, as expected under a Hill–Robertson interference scenario, where selected mutations reduce the effective population size at linked loci (Hill and Robertson 1966; Marais and Charlesworth 2003).

**Figure 4:**
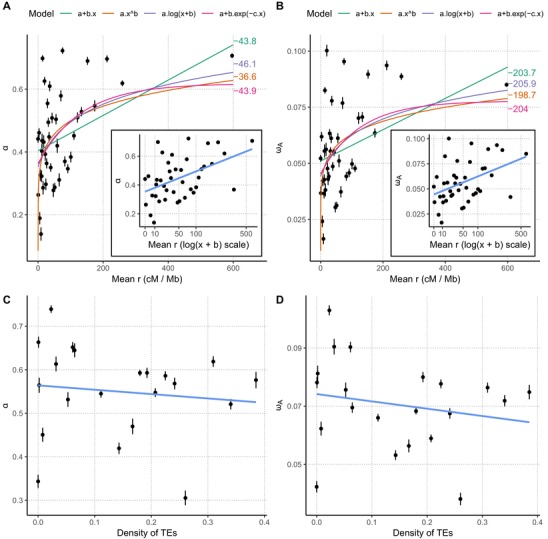
Estimates of the proportion of adaptive substitutions α, and the rate of adaptive substitution, ω_a_ as a function of the recombination rate (r). (A) α as a function of the *r*. (B) ω_a_ as a function of *r*. Each point and bars represent the mean estimate, and corresponding standard error for one recombination category over 100 bootstrap replicates. Four models were fitted (colored curves) and corresponding Akaike's information criterion values are indicated in the right margin. Inset plots represent the same data with a logarithmic scale; the b value was set to the corresponding estimate in the third model. Confidence intervals have been omitted for clarity. (C) and (D) Show the effect of TE density on α and ω_a_, respectively.

We further explored the relationship among α, ω_a_, and *r*. Without Hill–Robertson interference, the rate of adaptive substitution becomes independent of the recombination rate. The presence of an asymptote can therefore be used to assess the rate of adaptive substitutions in the absence of interference, and further quantify its impact on the rate of adaptation (Castellano et al. 2016). We fitted four models: linear (as in Campos et al. 2014), power law, curvilinear (as in Castellano et al. 2016), and logarithmic (see Materials and Methods). While we find higher support for the logarithmic model (Fig. 4), the effect is weak. When using *ρ* instead of *r*, the power law offers a better fit (Supporting Information). These results do not allow us to conclude confidently for or against the existence of an asymptotic value (Castellano et al. 2016).

As several authors (e.g., Ma et al. 2010; Raffaele et al. 2010; Rouxel et al. 2011; de Jonge et al. 2013) reported that TEs are associated with effector genes in several species and suggested that they play a role in adaptation, we tested whether the rate of adaptive substitutions was correlated with the amount of TEs in the vicinity of the genes. We stratified the dataset according to the local density of sites annotated as belonging to a TE (see Methods and Materials) and computed both α and ω_a_ for each category. We did not find a significant correlation among α, ω_a_, and the density of TEs (Kendall's tau = 0.0958, *P*‐value = 0.4293 for α and tau = 0.0824, *P*‐value = 0.4983 for ω_a_, Fig. 2C, D). These results, therefore, are not consistent with a positive role of TEs on the adaptive rate of proteins in the genome of *Z. tritici*.

## Conclusions

In this study, we have used the fungal wheat pathogen *Z. tritici* to assess the impact of sexual recombination on the patterns of selection on the genome of this rapidly evolving organism. Our results suggest a widespread occurrence of linked selection (both background selection and Hill–Robertson interference), as both the strength of negative selection and the rate of adaptation correlate with the recombination rate. The rate of adaptive substitutions in this plant pathogen, however, is similar to estimates in animal species and considerably faster than corresponding estimates in plants. Notably, rates of evolution in genes encoding effector proteins are more than twice as fast as the genome average, consistent with the essential role of these genes in host‐pathogen interactions. The recombination landscape of *Z. tritici* is highly heterogeneous, and the genes contributing to the high rate of adaptive evolution are located in regions of high recombination. That such high rates of adaptive substitutions are achieved in the presence of pervasive linked selection due to high densities of coding regions is remarkable, and highlight the fundamental role of recombination and sexual reproduction in adaptive processes of rapidly evolving organisms.

## Materials and Methods

### RE‐SEQUENCING, ASSEMBLY, AND ALIGNMENT OF *Z. tritici* ISOLATES

In this study, we used a geographical collection of 13 haploid field isolates of *Z. tritici* isolated from infected leaves of bread wheat (*Triticum aestivum*; Table S1). Genome data of three isolates, including the reference isolate IPO323, were published previously (Goodwin et al. 2011; Stukenbrock et al. 2011). For the remaining 10 isolates full genomes were sequenced. DNA extraction was performed as previously described (Stukenbrock et al. 2011). Library preparation and paired‐end sequencing using an Illumina HiSeq2000 platform were conducted at Aros, Skejby, Denmark. Sequence data of the 10 isolates have been deposited under the NCBI BioProject IDs PRJNA312067. We used SOAPdenovo2 (Luo et al. 2012) to construct de novo genome assemblies for each isolate independently. For each genome, the k‐mer value maximizing the N50 of contigs and scaffolds was selected.

Before generating a multiple‐genome alignment, we preprocessed the individual genomes of the 13 *Z. tritici* isolates. First, we masked repetitive sequences using a library of 497 repeat families de novo identified in four *Zymoseptoria* species (Grandaubert et al. 2015). Repeats were soft‐masked using the program RepeatMasker (option ‐xsmall) to retained information of repeat sites in the alignment (A.F.A. Smit, R. Hubley and P. Green RepeatMasker at http://repeatmasker.org). Second, we filtered the genome assemblies to contain only contigs with a length ≥1 kb. Multiple‐genome alignments were generated by the MULTIZ program using the LASTZ pairwise aligner from the Threaded Blockset aligner (TBA) package (Blanchette et al. 2004). The alignment was projected on the IPO323 reference genome using the maf_project program from the TBA package.

### PREDICTION OF EFFECTOR CANDIDATES

Gene models from the *Z. tritici* reference strains (Grandaubert et al. 2015) were used to predict proteins targeted for secretion using SignalP (Petersen et al. 2011). Genes predicted to encode a secreted protein were further submitted to effector prediction using the EffectorP software (Sperschneider et al. 2016).

### RECOMBINATION MAPS, DENSITIES OF CODING SITES, AND TRANSPOSABLE ELEMENTS

Local recombination rates in 20 kb windows were obtained from Croll et al. (2015) and averaged over the two crosses. Population recombination rates (*ρ*) in the same 20 kb windows were computed as in Stukenbrock and Dutheil (2017). Each gene was assigned a recombination rate based on the window(s) it overlapped with, using a weighted average in case it overlapped with multiple windows. Local protein‐coding site and TE densities were computed as the proportion of coding sites in a window starting x kb upstream and ending x kb downstream of each gene. We compared different estimations for x = 10, 20, 50 or 100 kb (see Supporting Information). For the density of coding sites, we find very little influence of the window size, with a unimodal distribution around ∼50%. We, therefore, selected the intermediate x = 50 kb. The density of TEs appeared to be 0‐inflated for low values of x. We, therefore, selected x = 100 kb in order to get a unimodal distribution.

### ESTIMATION OF RATES OF ADAPTATION

Based on the coordinates of each predicted gene model in the reference genome IPO323 (Goodwin et al. 2011; Grandaubert et al. 2015), exons were extracted from the multiple‐genome alignment of *Z. tritici* isolates using MafFilter (Dutheil et al. 2014). Complete coding sequences (CDS) were concatenated to generate individual alignments of all orthologous CDS. If one or more exons were not extracted from the alignment due to missing information, the gene was discarded from further analyses. Each complete CDS alignment was filtered according to the following criteria: (i) CDS were discarded if they contained more than 5% gaps in one or more individuals, (ii) CDS with premature stop codon were likewise deleted, and (iii) only alignments comprising three or more CDS were kept. In some cases, due to indels in the genome alignment, the codon phasing of some genes was lost. This issue was overcome by refining the CDS alignment using the codon‐based multiple alignment program MACSE (Ranwez et al. 2011). The final dataset contained 9412 gene alignments, among which 7040 contained a sequence for all 13 isolates. We further created a dataset containing an outgroup sequence, taken from the sister species *Z. ardabiliae*, leading to 6767 alignments with all 13 isolates together with the outgroup sequence.

The CDS alignment with outgroup was used to infer the synonymous and nonsynonymous divergence based on the rate of synonymous and nonsynonymous substitutions. The synonymous and nonsynonymous unfolded site frequency spectra (SFS) were computed, using the outgroup sequence to reconstruct the ancestral allele. To do so, we first reconstructed a BioNJ tree for each gene and fitted a codon model of evolution using maximum likelihood. Then ancestral state was inferred using the marginal reconstruction procedure of Yang et al. (1995). All calculations were performed using the BppPopStats program from the Bio++ Program Suite (Guéguen et al. 2013). We used the Grapes program in order to estimate the distribution of fitness effects from the SFS and compute a genome‐wide estimate of α and ω_a_, the proportion of mutations fixed by selection and the rate of adaptive substitutions, respectively (Galtier 2016). The following models were fitted and compared using Akaike's information criterion: Neutral, Gamma, Gamma‐Exponential, Displaced Gamma, Scaled Beta, and Bessel K. Analyses were conducted on the complete set of gene alignments, as well as on sub‐datasets sorted according to whether the individual genes encoded a predicted effector protein or not. To assess the significance of the difference between the inferred parameters in the two gene sets, 1000 permutations of genes between the two categories where generated, and parameters inferred independently on the two pseudo‐gene sets. We tested two statistics, S(α) = α(effectors) ‐ α(non‐effectors) and S(ω_a_) = ω_a_(effectors) – ω_a_(non‐effectors). We computed their respective *P*‐value using the formula $\left(\right|S\geq S_{obs}\left|+1)/(|S|+1\right)$, where |S|=1,000 is the number of permutations performed.

We further stratified our dataset according to the local recombination rate, computed in 20 kb windows, using both the previously published genetic maps (*r*, in cM/Mb) (Croll et al. 2015) and population estimates (*ρ* = 2.Ne.r) from patterns of linkage disequilibrium (Stukenbrock and Dutheil 2017). We discretized the observed distributions of both *r* and *ρ* in 41 and 45 categories, respectively, using the *cut2* command from the *Hmisc* R package in order to have a similar number of genes in each category (comprising between 247 and 258 genes for *ρ*, and between 67 and 1323 genes for *r*, the largest value being obtained for genes with *r* = 0). For each gene sets, 100 bootstrap replicates were generated by sampling genes randomly in each category. Genes in each replicate were pooled to compute site frequency spectra and the Grapes program run with the GammaExpo distribution of fitness effect (Galtier 2016). For each recombination category, the mean estimates of α and ω_a_, as well as the standard error over the 100 replicates, were computed after discarding all replicates with a negative estimate of α or ω_a_ (ranging from 5% to 10% of replicates discarded, depending on the category and analysis). We performed a similar analysis by discretizing the density of TEs in 100 kb windows.

### GENOME‐WIDE ANALYSIS OF SELECTION PATTERNS

We inferred the strength of purifying selection by computing the pN/pS ratio for each gene. Average pairwise synonymous (πS) and nonsynonymous (πN) nucleotide diversity were computed for each gene, and divided by the average number of synonymous (LS) and nonsynonymous (LN) positions, respectively, in order to compute the pN/pS ratio as (πN/LN)/(πS/LS). We compared the strength of purifying selection of each gene to several variables, after discarding six genes with pN/pS > 1, as they might be under positive selection. In addition to the local recombination rate, coding site density, and TE density, we recorded the GC content at the third codon position (GC3) and protein length. Expression levels were calculated from Kellner et al. (2014). The mean expression level was computed as the maximum value observed for the gene in axenic culture or plant infection; each averaged over three biological replicates. Genes located on accessory chromosomes were labeled as “dispensable.” Correlations of the pN/pS ratio with each explanatory variable were performed using rank‐based tests (Kendall correlation and Wilcoxon test), as implemented in the R statistical software.

### ESTIMATION OF CODON USAGE IN *Z*. *tritici*

We selected the 10% *Z. tritici* most expressed genes and computed the relative synonymous codon usage of every codon (Sharp et al. 1986). Analyses were conducted using the *uco* function of the *seqinr* package for R (Charif et al. 2005).

## CONFLICT OF INTEREST

The authors declare that they have no competing interests.

Associate Editor: S Wright

## Supporting information


**Fig S1**. Codon usage in *Z. tritici*. Relative synonymous codon usage (RSCU) in the 10% most expressed genes of *Z. tritici*. Codon usage, according to the base type at the third position.

**Table S1**. Summary table of isolates used in this study and genome assembly statistics.

**Table S2**. Summary statistics of the multiple‐genome alignment of thirteen *Z. tritici* genomes.
**Supplementary Data**. All scripts and data necessary to reproduce the analyses and figures have been deposited at FigShare under the https://doi.org/10.6084/m9.figshare.6848513.
