# Supplementary figures and images for: The genomic determinants of adaptive evolution in a fungal pathogen
Source: Evol Lett. 2019 May 1;3(3):299–312. doi: 10.1002/evl3.117 (PMC6546377; doi:10.1002/evl3.117)

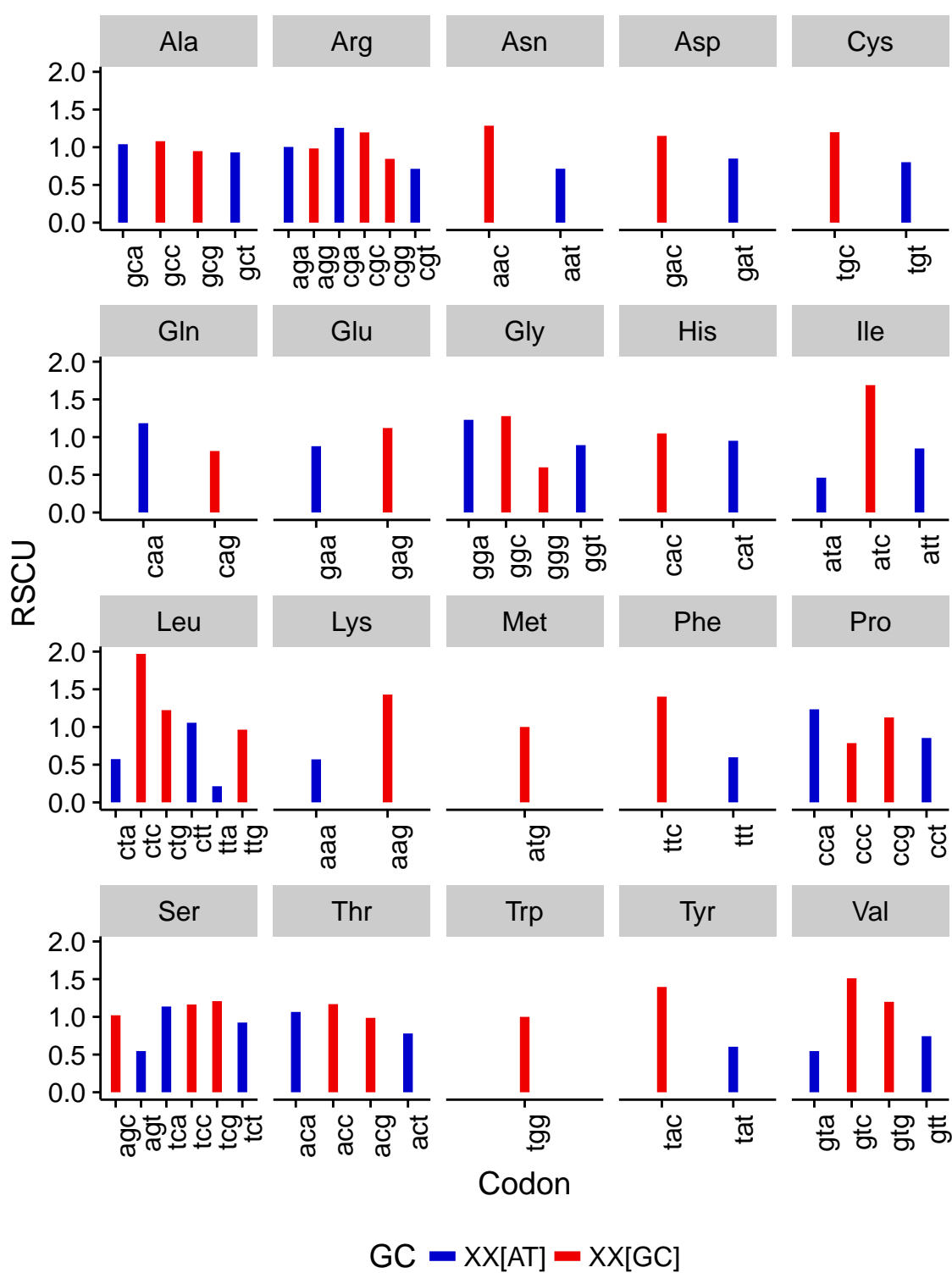

Supplement: Supplementary file 1 — Fig S1. Codon usage in Z. tritici. Relative synonymous codon usage (RSCU) in the 10% most expressed genes of Z. tritici. Codon usage, according to the base type at the third position. [file EVL3-3-299-s001.pdf]
